# Is Physical Activity Associated with Less Depression and Anxiety During the COVID-19 Pandemic? A Rapid Systematic Review

**DOI:** 10.1007/s40279-021-01468-z

**Published:** 2021-04-22

**Authors:** Sebastian Wolf, Britta Seiffer, Johanna-Marie Zeibig, Jana Welkerling, Luisa Brokmeier, Beatrice Atrott, Thomas Ehring, Felipe Barreto Schuch

**Affiliations:** 1 Department of Psychology, Institute of Clinical Psychology and Psychotherapy, University of Tuebingen, Tuebingen, Germany; 2 Institute of Sport Science, Department of Education & Health Research, University of Tuebingen, Tuebingen, Germany; 3 Mannheim Institute of Public Health, Mannheim Medical Faculty, University of Heidelberg, Mannheim, Germany; 4 Department of Psychology, LMU Munich, Munich, Germany; 5 Department of Sports Methods and Techniques, Federal University of Santa Maria, Santa Maria, Brazil

## Abstract

**Background:**

The Covid-19 pandemic is affecting the entire world population. During the first spread, most governments have implemented quarantine and strict social distancing procedures. Similar measures during recent pandemics resulted in an increase in post-traumatic stress, anxiety and depression symptoms. The development of novel interventions to mitigate the mental health burden are of utmost importance.

**Objective:**

In this rapid review, we aimed to provide a systematic overview of the literature with regard to associations between physical activity (PA) and depression and anxiety during the COVID-19 pandemic.

**Data Source:**

We searched major databases (PubMed, EMBASE, SPORTDiscus, and Web of Science) and preprint servers (MedRxiv, SportRxiv, ResearchGate, and Google Scholar), for relevant papers up to 25/07/2020.

**Study Eligibility Criteria:**

We included observational studies with cross-sectional and longitudinal designs. To qualify for inclusion in the review, studies must have tested the association of PA with depression or anxiety, using linear or logistic regressions. Depression and anxiety must have been assessed using validated rating scales.

**Study Appraisal and Synthesis Methods:**

Effect sizes were represented by fully adjusted standardized betas and odds ratios (OR) alongside 95% confidence intervals (CI). In case standardized effects could not be obtained, unstandardized effects were presented and indicated.

**Results:**

We identified a total of 21 observational studies (4 longitudinal, 1 cross-sectional with retrospective analysis, and 16 cross-sectional), including information of 42,293 (age 6–70 years, median female = 68%) participants from five continents. The early evidence suggests that people who performed PA on a regular basis with higher volume and frequency and kept the PA routines stable, showed less symptoms of depression and anxiety. For instance, those reporting a higher total time spent in moderate to vigorous PA had 12–32% lower chances of presenting depressive symptoms and 15–34% of presenting anxiety.

**Conclusion:**

Performing PA during Covid-19 is associated with less depression and anxiety. To maintain PA routines during Covid-19, specific volitional and motivational skills might be paramount to overcome Covid-19 specific barriers. Particularly, web-based technologies could be an accessible way to increase motivation and volition for PA and maintain daily PA routines.

## Key Points

**Table:** 

The Covid-19 pandemic increased symptoms of anxiety and depression symptoms. Those reporting a higher total time spent in moderate to vigorous physical activity, had 12–32% lower chances of presenting depressive symptoms and 15–34% of presenting anxiety.
The promotion of physical activity habits and routines might be a cost-effective and comprehensive worldwide applicable strategy to overcome the severe gap between people in need and people receiving mental health care, especially in low-income countries with even non-existing mental health supplies.
Web-based technologies might be promising tools to increase motivation and volition for PA and maintain daily physical activity routines even under pandemic-specific barriers. However, there is a clear need for more systematic research for effectively and safely usable apps or web-based programs to prevent psychiatric disorders through physical activity.

## Introduction

With 106,125,682 confirmed cases all over the world (up to February 10th, 2021), COVID-19 is a global public health emergency. COVID-19 is characterized by a fast human-to-human transmission through droplet or close contact. Given the lack of appropriate treatments and vaccines during the early stage of the pandemic, many countries implemented procedures recommended by the World Health Organization (WHO) [1], such as the isolation of symptomatic patients, quarantining individuals with the history of contact with COVID-19 infected persons, and further anti-contagion policies such as mandatory stay at home or business closures. Those anti-contagion policies substantially reduced exponential growth rates [2, 3].

Quarantine and social distancing measures had already been successfully enforced during earlier pandemics, such as the 2003 outbreak of SARS and the 2014 outbreak of Ebola [4]. However, studies on the effects of these measures have reported elevated symptoms of anxiety, post-traumatic stress, and depressive disorders, as well as a 30% higher suicide rates in populations impacted by these measures [5, 6]. These findings are being replicated during the Covid-19 pandemic with multiple studies reporting elevated prevalences of depression and anxiety [7–11].

Notably psychiatric disorders result in a considerable burden of disease, accounting for 6.7% of overall disability-adjusted life years [12] and being attributable to 14.3% of death worldwide [13]. Despite the high burden of psychiatric disorders, there is a severe gap between people in need and people receiving mental health care [14]. This general treatment gap is especially severe in low- and middle-income countries, where 76–85% of people with mental disorders do not receive any treatment [15]. The latest WHO “mental health Atlas” indicates that only 95.6 out of 100 000 depressed cases worldwide receive any professional mental health care, whereas the treatment prevalence in high-income countries is 16-times higher compared to low-income countries [16]. Although there is no current global data available, the treatment gap is assumed to be much higher during or after the Covid-19 pandemic. Access to general mental health care might be restricted for several reasons, including supply priorities that being focused on Covid-19 infections, medication shortages, prohibition of face-to-face psychotherapeutic sessions of psychological treatment, closing of inpatient facilities to mention only some reasons. Indeed, current international position papers point out a clear need to adapt and improve mental health services worldwide due to these specific challenges during and after the pandemic [17, 18]. To mitigate the negative mental health consequences of pandemics, evidence suggests that policymakers should ensure quarantine measures to be as short as possible, to provide adequate general supplies for basic needs, give people as much information as possible and strengthen social support and communication among people affected by the pandemic [4]. A recently published position paper on research priorities for mental health science regarding COVID-19 [18] demands the interdisciplinary development of novel interventions to protect mental wellbeing by mechanistically based approaches to strengthen altruism and prosocial behavior. Among others, physical activity (PA) interventions are highlighted as a promising approach. PA is defined as any bodily movement produced by skeletal muscles that results in energy expenditure and exercise is defined as PA, which is planned, structured, and repetitive, with the primary aim to improve or maintain physical fitness [19]. International PA guidelines recommend 150 min of moderate or 75 min of vigorous intensity PA per week for optimal physical and mental health benefits [20]. Indeed, in pre-pandemic times PA has been identified as a protective factor against incident depression [21] and anxiety [22]. However, decreased levels of PA were observed in the general population in multiple countries [11, 23, 24] during the pandemic. This rapid systematic review aims to outline current evidence regarding the associations of PA and exercise with depression and anxiety during the Covid-19 pandemic.

## Methods

In this rapid review, we sought for observational studies examining the associations of PA and depression and anxiety during the COVID-19 pandemic. Inclusion criteria were: (1) observational studies in any population, including cross-sectional and longitudinal designs. Longitudinal studies could be either prospective or retrospective; (2) studies have tested the association of PA with depression or anxiety, using linear or logistic regressions; (3) depression and anxiety were assessed using validated screening or diagnostic tools. We excluded opinion pieces, systematic reviews, and studies addressing other viruses.

We searched the electronic databases PubMed, EMBASE, SPORTDiscus, and Web of Science using the following strategy: (physical activity OR exercise OR sport) AND (coronavirus OR sars-cov-2 OR COVID* OR severe acute respiratory syndrome OR pandemic) AND (depression OR anxiety OR mental health). Preprints were searched in MedRxiv, SportRxiv, and SciELO Preprints using the following strategy: “(physical activity OR exercise) AND (coronavirus OR sars-cov-2 OR COVID* OR severe acute respiratory syndrome OR pandemic)”. Additional hand searches were performed on COVID-19 online repositories on ResearchGate and Google Scholar. Searches were made by an experienced reviewer (FS) on 29th July, 2020. Study selection was conducted in three steps: (1) duplicates removal; (2) screening at the title and abstract level; and (3) assessment based on full-text. The selection was made by one reviewer (FS). Data extraction of selected studies was then performed by three researchers (FS, BS, SW). Data extracted were: author and year, country of the included sample, study design, sample size, age group of the sample included, when possible, mean or range of age sample, % of women, instrument/question used to assess PA levels, instruments used to assess depression and anxiety, publication type and statistical outcomes (regression standardized beta coefficients and odd’s ratios). If they were indicated in the report, fully adjusted coefficients and odd’s ratios were extracted. As studies included in this review used very heterogeneous statistical approaches, a meta-analysis could not be conducted. Instead, we summarized the evidence and presented effect sizes [betas and odds ratios (OR)] with confidence intervals and indicated significant associations between PA and depression or anxiety, separately (see Table 2). In case the study just reported the unstandardized betas, we requested the standardized betas by e-mail. If standardized effects could not be obtained, unstandardized effects were presented and indicated. The risk of bias of individual studies was assessed using the National Institutes of Health (NIH) study quality assessment tool for observational cohort and cross-sectional studies [25]. The NIH tool assessment is composed by 14 questions the risk of potential selection bias, information bias measurement bias or confounding bias. There are three options (yes, no, other) for each question. Each “no” or “other” is suggestive of the presence of some risk of bias. Questions #6 (exposure prior outcome), #7 (sufficient time to see an effect), #10 (repeated exposure assessment), and #13 (follow-up rate) were disregarded for cross-sectional studies. Due to the self-reported nature of the assessments, question #12 (blinding of outcome assessors) was also disregarded for all studies.

## Results

**Fig. 1:**
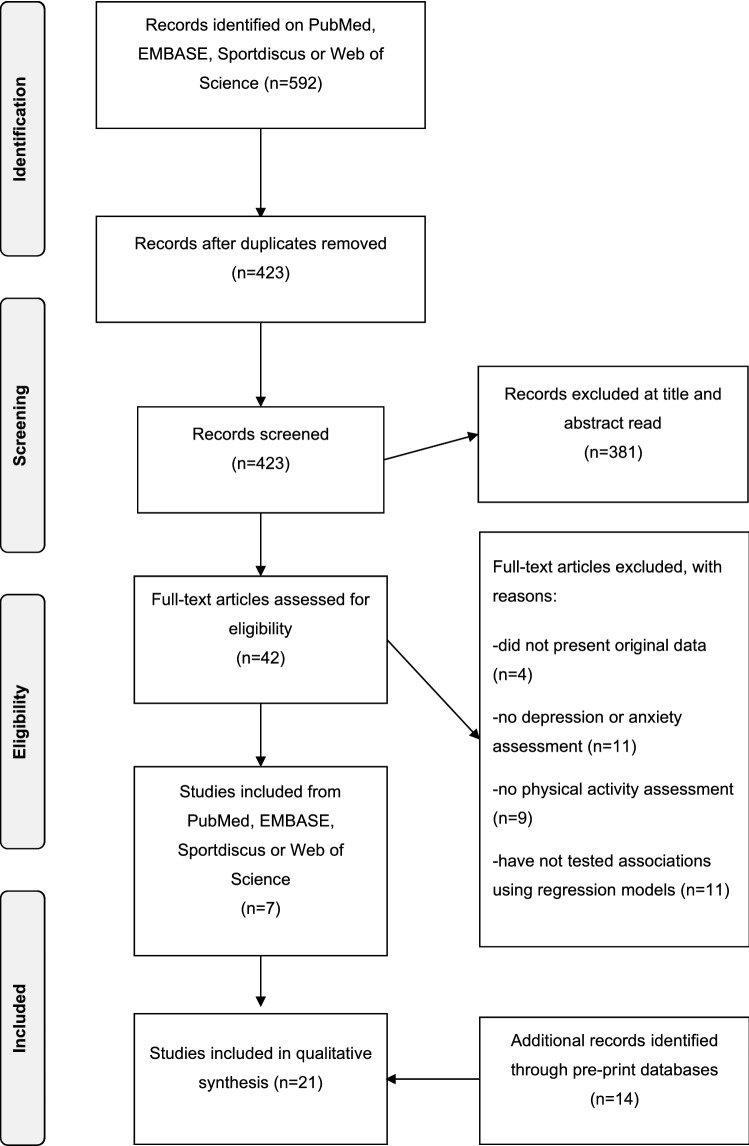
PRISMA flow-chart of the screening and selection of studies

Searches on PubMed, EMBASE, Sportdiscus, and Web of Science resulted in 592 potentially relevant studies. Preprint databases identified additional 572 potentially relevant studies. A flow-chart of the selection process is provided in Fig. 1. Of the identified studies, 21 studies meet the criteria [7, 8, 11, 26–43]. Four studies had a prospective longitudinal design [29, 32, 39, 42], 1 was a cross-sectional study with a retrospective measure of the exposure factor (henceforth treated as retrospective) [7], and 16 were cross-sectional studies [8, 11, 26–28, 30–38, 40, 41, 43, 44]. A total of 7 studies were conducted in Asia [27, 28, 30, 33, 36, 42, 43], 6 in Europe [11, 29, 34, 35, 38, 39], 3 in South America [8, 31, 32], 3 in North America [7, 26, 37], 1 in Oceania [41] and 1 study included a multinational sample [40].

Data form a total of 42,293 (median = 68% of women) participants were included. Only 1 study was exclusively composed by older adults (over 50 years), 4 were in children, adolescents, or young adults, while 13 studies were in adults (over 18). Only 7 studies used validated measures to assess PA levels. A wide range of scales to measure depression or anxiety were used, the most used scales being the Beck Depression and Anxiety inventory and the DASS-21. Most studies (*n* = 14) were peer-reviewed, seven studies were published as preprints. A summary of studies is provided in Table 1.

**Table 1:** Characteristics of included studies

Author	Country	Design	Type	*N*	Age group (years)		% females	PA assessment	MH assessment
Bauer et al. [11]	Germany	Cross-sectional	Preprint	3,700	Adults (*M* = 33.13)		78.6	BSA-F	PHQ-9; GAD-7
Callow et al. [26]	US	Cross-sectional	Peer-reviewed	1,046	Older adults (< 50)		80	PASE	GDS; GAS
Chen et al. [27]	China	Cross-sectional	Peer-reviewed	1,036	Children/adolescents (*R* = 6–15)		48.7	NR	DSRS-C; SCARED
Chen et al. [28]	Iran	Cross-sectional	Preprint	474	Adults (*R* = 20–70)		51.3	Single item (hours/day)	PHQ-2; GAD-2
Cheval et al. [29]	France, Switzerland	Longitudinal (retrospective and prospective)	peer-Reviewed	110	Adults (*M* = 43)		68	IPAQ	PROMIS (adapted questions for depression and anxiety)
Deng et al. [30]	China	Cross-sectional	Peer-reviewed	1,607	Adolescents/young adults (NR)		35.2	Multiple items (duration, frequency)	DASS-21
Filgueiras and Stultz-Kolehmainen [32]	Brazil	Cross-sectional	Preprint	1,460	Adults (*M* = 32.9)		72.87	Single item (frequency)	FDI; SSTAI
Filgueiras and Stultz-Kolehmainen [31]	Brazil	Longitudinal (prospective)	Preprint	360	Adults (*M* = 37.9)		68.8	Multiple items (frequency, type)	FDI; SSTAI
Fu et al. [33]	China	Cross-sectional	Peer-reviewed	1,242	Adults (NR)		69.7	NR	PHQ-9; GAD-7
Fullana et al. [34]	Spain	Cross-sectional	Peer-reviewed	5,545	Adults (*M* = 47)		73	NR	PHQ-9; GAD-7
Jacob et al. [35]	UK	Cross-sectional	Peer-reviewed	902	Adults (NR)		63.8	Multiple items (duration/day, intensity)	BDI; BAI
Khan et al. [36]	Bangladesh	Cross-sectional	Peer-reviewed	505	Adolescents/young adults		37.3	NR	DASS-21
Lebel et al. [37]	Canada	Cross-sectional	Peer-reviewed	1,987	Adults (*M* = 32.4)		100	Godin Shephard Leisure-Time Exercise Scale	EPDS; PROMIS anxiety
Meyer et al. [7]	US	Longitudinal (retrospective)	Peer-reviewed	3,052	Adults (NR)		62	Multiple items (duration/day, intensity)	BDI; BAI
Moreira et al. [38]	Portugal	Cross-sectional	Preprint	1,280	Adults (*M* = 37.1)		79.8	Single item (duration/day, intensity)	DASS-21
Planchuelo-Gómez et al. [39]	Spain	Cross-Sectional	PEER-reviewed	1,056	Adults (*M* = 32.1)		67.6	NR	DASS-21
Plomecka et al. [40]	Multiple (12 countries)	Cross-sectional	Preprint	12,817	Adults (NR)		72.3	NR	BDI
Schuch et al. [8]	Brazil	Cross-sectional	Peer-reviewed	937	Adults (NR)		72.3	Multiple items (duration/day, intensity)	BDI; BAI
Stanton et al. [41]	Australia	Cross-sectional	Peer-reviewed	1,491	Adults (*M* = 50.5)		67	AAS	DASS-21
Zhang et al. [42]	China	Longitudinal (prospective)	Peer-reviewed	66	Adolescents/adults (*M* = 20.7)		62.1	IPAQ	DASS-21
Zheng et al. [43]	China	Cross-sectional	Preprint	1,620	Children/adolescents (*M* = 10.1)		47.8	Single item (decrease since Covid)	DSRS-C; SASC

*AAS* Active Australia Survey, *BASF-F* The Physical Activity Exercise, and Sport Questionnaire, *DASS-21* Depression and Anxiety Scale 21 items, *DSRS*-C Depression Self-Rating Scale for Children, *EPDS* Edinburgh Depression Scale, *FDI* Filgueira depression inventory, *GAD-7* Generalized Anxiety Screener 7, *GAS* Geriatric Anxiety Scale, *GDS* Geriatric Depression Scale, *IPAQ* International Physical ACTIVITY Questionnaire, *M* mean, *MH* mental health, *n *number of participants, *NR* not reported, *PA *physical activity, *PAVS* physical activity vital sign, *PASE* Physical Activity Scale for the Eldery, *PHQ-9* Patient Health Questionnaire 9, *PROMIS* Patient-Reported Outcomes Measurement Information System, *R* range, *SASC* Social Anxiety Scale for Children, *SCARED* Screen for Child Anxiety Related Disorders, *SSTAI* The Spielberg State and Trait Anxiety Inventory

Results are summarized and presented in Table 2. Out of ten studies reporting analyses on the association between the overall volume of PA and depression, seven studies showed that more PA is significantly associated with less depression symptoms [26, 28, 30, 35, 36, 38, 40], and three out of nine studies investigating the association between the overall volume of PA and anxiety symptoms showed that more PA is significantly associated with less anxiety symptoms [28, 35, 38]. Three out of six studies reported higher frequencies of PA to be significantly associated with less depression [30, 32, 39] and two out of five studies to be significantly associated with less anxiety [30, 32]. One study showed that vigorous but not moderate PA is significantly associated with less depression and anxiety symptoms [8] and another study indicated that light and vigorous PA is significantly correlated with less depression, but moderate intensity was not [26]. Out of four studies assessing an association between regular and guideline-consistent PA and depression and anxiety symptoms, two studies demonstrate that regular PA (compared to not regular) is significantly associated with less depression and anxiety symptoms [30, 33]. One study demonstrated that guideline conforming moderate to vigorous PA is associated with lower odds of depression and anxiety [8]. Five out of six studies showed that a decrease in PA during the pandemic was significantly associated with more depression symptoms [7, 11, 30, 41, 43] and three out of six studies showed that a decrease in PA was significantly associated with more anxiety symptoms [11, 30, 41]. One study reported that an increase in PA was associated with less depressive symptoms [42].

**Table 2:** Main results of multiple linear and logistic regressions analyzing the association of physical activity or exercise with symptoms of depression or anxiety in the included studies

Author		Predictor	Depressive symptoms			Anxiety symptoms	
			Beta (95% CI)	OR (95% CI)		Beta (95% CI)	OR (95% CI)
**Volume**							
Bauer et al. [11]		EX (minutes/week)	0.00# (NR; NR)			0.01# (NR; NR)	
Callow et al. [26]		PA (PASE score)	− 0.22*** (NR; NR)			− 0.02 (NR; NR)	
Chen et al. [28]		EX (hours/day)		0.68* (0.47; 0.97)			0.66* (0.45; 0.96)
Deng et al. [30]		EX (> 60 min/day; Ref: < 60 min/day))	− 0.08*** (NR;NR)			− 0.05 (NR; NR)	
Cheval et al. [29]		PA (minutes/day)	NR^#^ (NR; NR)			NR^#^ (NR; NR)	
Jacob et al. [35]		EX (minutes/day)		0.88° (0.8; 0.97)			0.85° (0.79; 0.97)
Khan et al. [36]		EX (any amount;	− 2.1* (− 4.02; − 0.17)^a^			− 0.55 (− 1.92; 0.82)^a^	
		Ref.: No EX)					
Moreira et al. [38]		EX (hours)	− 1.17° (NR; NR)^a^			− 0.81° (NR; NR)	
Plomecka et al. [40]		EX (> 15 min/day;	− 0.13*** (NR; NR)	NR^#^ (NR; NR)			
		Ref.: ≤ 15 min/day, < 60 min/day)					
		EX (≥ 60 min/day;	− 0.15*** (− 0.18; − 0.12)	NR^#^ (NR; NR)			
		Ref.: ≤ 15 min/day)					
Schuch et al. [8]		PA (minutes/day; per 10 min increase)	− 0.03 (− 0.1; 0.03)			− 0.05 (− 0.13; 0.02)	
**Frequency**							
Deng et al. [30]		EX (1 to 2 times/week; Ref: < 1x/week)	− 0.11*** (NR; NR)			− 0.09** (NR; NR)	
		EX (> 2 times/week; Ref: < 1x/week)	− 0.15*** (NR; NR)			-0.12** (NR; NR)	
		EX (every day; Ref: < 1x/week)	− 0.11*** (NR; NR)			-0.09* (NR; NR)	
Filgueiras and Stultz-Kolehmainen [32]		EX (frequency/week)	− 2.68** (NR; NR)^a^			− 1.64*** (NR; NR)^a^	
Fullana et al. [34]		EX (Unclear)		0.93 (NR; NR)			0.95 (NR; NR)
Lebel et al. [37]		EX (Godin Shephard Leisure-Time Exercise Score)	− 0.01* (NR; NR)	0.99 (0.99; 0.99)		− 0.01** (NR; NR)	0.99 (0.99; 1.0)
Planchuelo-Gómez et al. [39]		EX (1–2 times/week; Ref.: No EX)	− 0.17 (NR; NR)^a^				
		EX (3–5 times/week; Ref.: No EX)	− 0.85* (NR; NR)^a^				
		EX (6–7 times/week; Ref.: No EX)	− 1.29*** (NR; NR)^a^				
Filgueiras and Stultz-Kolehmainen [31]		EX (frequency/week)	NR^#^ (NR; NR)			NR^#^ (NR; NR)	
**Intensity**							
Callow et al. [26]		Light PA (PASE score)	0.12** (NR; NR)				
		Moderate PA (PASE score)	− 0.01 (NR; NR)				
		Vigorous PA (PASE score)	0.09* (NR; NR)				
Schuch et al., 2020 [8]		Vigorous PA (minutes/day)	− 0.19* (− 0.34; − 0.04)	0.6** (0.44; 0.83)		− 0.22* (− 0.4; − 0.03)	0.71** (0.52; 0.96)
		Moderate PA (minutes/day)	0.00 (− 0.09; 0.09)	0.77 (0.57; 1.02)		− 0.03 (− 0.14; 0.08)	0.75 (0.58; 1)
**Regular/guideline conforming**							
Chen et al. [27]		EX (regular;		0.37 (NR; NR)^b^			0.43 (NR; NR)^b^
		Ref.: not regular)					
Deng et al. [30]		EX (regular;	− 0.2*** (NR; NR)			− 0.14*** (NR; NR)	
		Ref.: not regular)					
Fu et al. [33]		EX (not regular;		1.71*** (1.28; 2.29)			1.45* (1.08; 1.93)
Schuch et al. [8]		PA (≥ 30 min/day; Ref.: < 30 min/day)		0.72* (0.54; 0.96)			0.72* (0.54; 0.96)
**Change**							
Bauer et al. [11]		EX (less; equal; more)^c^	− 0.08*** (NR; NR)			− 0.05*** (NR; NR)	
Deng et al. [30]		EX (no change; Ref.: large change)	− 0.27*** (NR; NR)			− 0.21*** (NR; NR)	
		EX (little change; Ref.: large change)	− 0.22*** (NR; NR)			− 0.17*** (NR; NR)	
Filgueiras et al. [31]		EX (none, increase, decrease)	NR^#^ (NR; NR)			NR^#^ (NR; NR)	
Meyer et al. [7]		PA (increased; Ref.: maintained high)	− 0.01 (− 0.05; 0.02)			0.00 (− 0.03; 0.04)	
		PA (decreased; Ref.: maintained high)	0.09*** (0.05; 0.13)			0.03 (− 0.01; 0.07)	
		PA (maintained low; Ref.: maintained high)	0.04 (0.00; 0.07)			0.02 (− 0.02; 0.05)	
Stanton et al. [41]		PA (negative change; Ref.: no change/positive change)		1.08*** (1.06; 1.11)			1.09*** (1.05; 1.13)
Zhang et al. [42]		PA (per 100 MET increase)	− 0.04* (− 0.08; 0)			− 0.03 (− 0.07; 0)	
Zheng et al. [43]		PA (decrease vs. no change/increase)		2.07** (NR; NR)			1.24 (NR; NR)

*AOR* adjusted odd's ratio, *EX* exercise, *MET* metabolic equivalent of tasks, *NR* not reported, *OR* odd's ratio, *PA* physical activity, *PASE* Physical activity Scale for the Elderly, *Ref.* reference category

*p < 0.05; **p < 0.01; ***p < 0.001; °significant association, *p* value not reported; ^#^no significant association, *p* value not reported

^a^Unstandardized regression coefficient

^b^Odd's ratio calculated from case counts

^c^Post hoc analysis revealed that a decrease in exercise was significantly associated with less depression compared to stable exercise and increase. No other comparison reached significance

The risk of bias of individual studies is presented in Table 3. All studies clearly defined their research questions and used valid tools to assess main outcomes. Among the cross-sectional studies, 11 (68.75%) studies did not report the participation rate or included less than 50% of eligible participants, and 13 (81.25%) did not use valid tools to assess the exposure measure. A total of three out of five (60%) longitudinal studies are at risk of bias in the evaluating the definition of the study population, the participation rate, the validity of the exposure measure and in the retention of the sample.

**Table 3:** Risk of bias assessment (NIHM tool for observational studies)

Items	1	2	3	4	5	6	7	8	9	10	11	12	13	14
Cross-sectional studies														
Bauer et al. [11]	Y	Y	Y	Y	Y	–	–	Y	Y	–	Y	–	–	Y
Callow et al. [26]	Y	Y	Y	Y	Y	–	–	Y	Y	–	Y	–	–	Y
Chen et al. [27]	Y	N	NR	N	Y	–	–	N	N	–	Y	–	–	N
Chen et al. [28]	Y	Y	N	N	Y	–	–	Y	N	–	Y	–	–	Y
Deng et al. [30]	Y	Y	Y	Y	N	–	–	Y	N	–	Y	–	–	N
Filgueiras and Stultz-Kolehmainen [32]	Y	N	NR	Y	Y	–	–	Y	N	–	Y	–	–	Y
Fu et al. [33]	Y	Y	NR	Y	Y	–	–	N	N	–	Y	–	–	Y
Fullana et al. [34]	Y	Y	NR	Y	N	–	–	N	N	–	Y	–	–	Y
Jacob et al. [35]	Y	Y	NR	Y	Y	–	–	Y	N	–	Y	–	–	Y
Khan et al. [36]	Y	N	NR	Y	Y	–	–	N	N	–	Y	–	–	Y
Lebel et al. [37]	Y	Y	NR	Y	Y	–	–	N	N	–	Y	–	–	Y
Moreira et al. [38]	Y	Y	NR	Y	Y	–	–	N	N	–	Y	–	–	Y
Plomecka et al. [40]	Y	Y	Y	Y	Y	–	–	Y	N		Y			Y
Schuch et al. [8]	Y	Y	NR	Y	Y			Y	N	–	Y	–	–	Y
Stanton et al. [41]	Y	Y	NR	Y	Y	–	–	N	Y	–	Y	–	–	Y
Zheng et al. [43]	Y	Y	Y	Y	N	–	–	N	N	–	Y	–	–	Y
**Longitudinal studies**														
Cheval et al. [29]	Y	N	Y	Y	Y	Y	Y	Y	Y	Y	Y	–	Y	NR
Filgueiras and Stultz-Kolehmainen [31]	Y	N	NR	Y	Y	Y	Y	Y	N	Y	Y	–	N	Y
Meyer et al. [7]	Y	Y	Y	Y	Y	N	Y	Y	N	N	Y	–	NA	Y
Planchuelo-Gómez et al. [39]	Y	Y	NR	Y	N	Y	Y	Y	NR	Y	Y	–	N	Y
Zhang et al. [42]	Y	N	NR	Y	Y	Y	Y	Y	Y	Y	Y	–	Y	NR

*Y* yes, *N* no, *NR* not relevant

## Discussion

The present study is, to the best of our knowledge, the first study to summarize the evidence on the associations of PA with depression and anxiety during the COVID-19 pandemic. The majority of studies included in the present review showed that those who performed PA on a regular basis with higher volume and frequency and kept the PA routines stable, showed less symptoms of depression and anxiety. There was consistent evidence that those who could not keep their PA routine stable during the pandemic showed more depression and anxiety symptoms [7, 11, 30, 41–43]. However, the association was more consistent regarding depressive compared to anxiety symptoms. Those reporting a higher total time spent in moderate to vigorous PA had 12% to 32% lower chances of presenting depressive symptoms and 15–34% of presenting anxiety. These findings are in line with results of recent meta-analyses showing that those with higher PA levels were 17% less likely of developing depression [21] and 26% less likely to develop anxiety [22] independently of the COVID-19 pandemic.

Indeed, the observed reduction in PA behavior during COVID-19 specific conditions is highly expected. For example, due to social distancing, exercising in a group setting was limited or completely prohibited. However, high social support is associated with more engagement in PA [45]. Indeed, social support was one of the strongest factors associated with adherence to PA in effective exercise interventions [46]. Furthermore, the COVID-19 pandemic impaired opportunities to be physically active due to the closure of sports clubs, gyms, or common indoor and outdoor places for PA. While some people were still allowed to do exercises like jogging on the streets, others were not [47]. In general, a lack of sporting opportunities seems to be associated with reduced PA [48]. Further negative consequences of the pandemic such as financial insecurities might have caused stress in individuals and stress, in turn, may differentially impact individuals’ level of PA. Whereas habitually active individuals might even increase their level of PA, those who had not yet integrated exercise as a part of daily life, reduce their level of PA [49]. Thus, habitually active individuals might have built PA-related health competence and learned to utilize PA as a strategy to cope with negative feelings, such as stress, that may arise with sudden adaptions [50, 51]. Therefore, to prevent an increase in psychiatric disorders during the current or further pandemics, factors that facilitate the integration of PA into daily life routines, such as motivational and volitional skills, need to be identified and encouraged. Motivation and volition are core components of several theories of behavior change such as the Health Action Process Approach (HAPA) [52]. HAPA is a social-cognitive model specifying motivational and volitional determinants of health behavior such as building intentions for health behavior, planning the behavior, coping with specific challenges, maintaining the behavior, and perceiving self-efficacy for all processes. A recent meta-analysis shows that action self-efficacy has large effects on health behavior through intentions and maintenance self-efficacy [53]. Especially, self-efficacy in building intentions and action planning have larger effects on physical activity behavior compared with other health behavior [53]. Indeed, Covid-19 specific interventions should even more focus on self-efficacy experiences in building intentions for PA and performing PA, since PA routines are interrupted through anti-contagion policies. A widely used way to promote these motivational and volitional determinants is the application of behavior change techniques (BCTs) [54, 55]. During the COVID-19 pandemic, some BCTs appear to be particularly important for the maintenance of regular PA. For instance, the knowledge about the benefits of PA on symptoms that accompany lock-down procedures, such as lowered mood or anxiety might strengthen intentions for PA [4–6]. Furthermore, individuals need the strong ability of coping planning to anticipate barriers that could discourage them to engage in PA (e.g., curfew, closed facilities) and find strategies to overcome them (e.g., engage in home training).

A web-based tool, e.g., a smartphone application could be a low-threshold and cost-effective option to train, supervise, apply, and adopt such BCTs, especially in terms of COVID-19. First empirical evidence showed preliminary efficacy of apps in promoting PA. Users of such apps are more likely to meet recommendations on PA than non-users [56–59]. However, the evidence of long-term effects is currently inconclusive, since only few studies assess long-term effects. A current meta-analysis claims for more research to further elucidate the time course of intervention effects [59]. Furthermore, a meta-analysis showed that internet-delivered interventions, which are able to use different BCTs, were effective in increasing PA [60]. A major advantage of such web-based tools is the possibility to overcome some of the COVID-19 specific barriers. For instance, it is possible to become physically active online with friends or a virtual community, which might work against the lack of social support. In addition, limited sporting opportunities may be expanded through fitness technology and the provision of structured programs, as they can be used both indoors (e.g., through fitness videos) and outdoors (e.g., through running apps) and, therefore, be adapted to the specific situation.

## Limitations

Most of the studies included in this review used cross-sectional research designs. A causal nature of these associations, therefore, remains unclear. There are notably differences in effect sizes which point at a high heterogeneity of the effects. Several studies further showed methodological shortcomings, e.g., not reporting the participation rate, including less than 50% of eligible participants, no validated tools to assess PA and failure to report standardized coefficients. Heterogeneity in research designs and statistical analyses hindered meta-analytic approaches, which would have provided a more sophisticated overall effect estimate. Finally, several included studies were published as preprints and are currently in review processes for final publications. It is, therefore, planned to update this review in the future.

## Conclusions, Future Research Directions, and Implications

This rapid review shows promising evidence that higher volume and frequency of PA and the keeping of regular PA habits during the Covid-19 pandemic are associated with less symptoms of depression and anxiety. For instance, those reporting a higher total time spent in moderate to vigorous PA had 12–32% lower chances of presenting depressive symptoms and 15–34% of presenting anxiety. Thus, the promotion of PA habits and routines might be a cost-effective and comprehensive worldwide applicable strategy to overcome the severe gap between people in need and people receiving mental health care, especially in low-income countries with even non-existing mental health supplies. Particularly, web-based technologies, could be an easily accessible way to increase motivation and volition for PA and maintain daily PA routines even under pandemic-specific barriers. However, only very few apps or websites have been tested in RCTs with high methodological standards [59, 61]. Thus, there is a clear need for more systematic research for effectively and safely usable apps or web-based programs to prevent psychiatric disorders through PA.

## Declarations

### Availability of data and material

Data sharing not applicable to this article since all data extracted from studies are presented in the current paper.
